# Research and Education Needs for Complex Generics

**DOI:** 10.1007/s11095-021-03149-y

**Published:** 2021-12-24

**Authors:** Sydney Stern, Jill Coghlan, Vishalakshi Krishnan, Sam G. Raney, Andrew Babiskin, Wenlei Jiang, Robert Lionberger, Xiaoming Xu, Anna Schwendeman, James E. Polli

**Affiliations:** 1 Department of Pharmaceutical Sciences, University of Maryland, 20 Penn Street, Baltimore, Maryland 21201 USA; 2 Department of Pharmaceutical Sciences, University of Michigan, 428 Church Street, Ann Arbor, Michigan 48109 USA; 3 Food and Drug Administration, Center for Drug Evaluation and Research, Office of Generic Drugs, Office of Research and Standards, 10903 New Hampshire Avenue, White Oak, Maryland 20993 USA; 4 Food and Drug Administration, Center for Drug Evaluation and Research, Office of Pharmaceutical Quality, Office of Testing and Research, 10903 New Hampshire Avenue, White Oak, Maryland 20993 USA

**Keywords:** bioequivalence, complex generic, formulation, generic, survey

## Abstract

Complex generics are generic versions of drug products that generally have complex active ingredients, complex formulations, complex routes of delivery, complex dosage forms, are complex drug-device combination products, or have other characteristics that can make it complex to demonstrate bioequivalence or to develop as generics. These complex products (i.e. complex generics) are an important element of the United States (U.S.) Food and Drug Administration’s (FDA’s) Generic Drug User Fee Amendments (GDUFA) II Commitment Letter. The Center for Research on Complex Generics (CRCG) was formed by a grant from the FDA to address challenges associated with the development of complex generics. To understand these challenges, the CRCG conducted a “Survey of Scientific Challenges in the Development of Complex Generics”. The three main areas of questioning were directed toward which (types of) complex products, which methods of analysis to support a demonstration of bioequivalence, and which educational topics the CRCG should prioritize. The survey was open to the public on a website maintained by the CRCG. Regarding complex products, the top three selections were complex injectables, formulations, and nanomaterials; drug-device combination products; and inhalation and nasal products. Regarding methods of analysis, the top three selections were locally-acting physiologically-based pharmacokinetic modeling; oral absorption models and bioequivalence; and data analytics and machine learning. Regarding educational topics, the top three selections were complex injectables, formulations, and nanomaterials; drug-device combination products; and data analytics, including quantitative methods and modeling & simulation. These survey results will help prioritize the CRCG’s initial research and educational initiatives.

**Supplementary Information:**

The online version contains supplementary material available at 10.1007/s11095-021-03149-y.

## INTRODUCTION

The scientific and regulatory framework for developing generic versions of brand name drugs was established by the Drug Price Competition and Patent Term Restoration Act of 1984. These “Hatch–Waxman Amendments” established the approval pathway under which generic drug developers (applicants) can submit an abbreviated new drug application (ANDA) to the United States (U.S.) Food and Drug Administration (FDA) that would include certain types of information and evidence to support the approval of an ANDA. However, pharmaceutical science has advanced substantially during the decades since the Hatch–Waxman Amendments, and the scientific complexity of many modern drugs can make it challenging to establish pharmaceutical equivalence (PE) and/or bioequivalence (BE). The complexity of developing complex generics can create vulnerabilities for potential drug shortages, and can limit patient access to affordable, high quality medicines (1).

Per the Generic Drug User Fee Amendments (GDUFA) II Commitment Letter of 5/12/2016, complex products generally include: 1. Products with complex active ingredients (e.g., peptides, polymeric compounds, complex mixtures of APIs, naturally sourced ingredients); complex formulations (e.g., liposomes, colloids); complex routes of delivery (e.g., locally acting drugs such as dermatological products and complex ophthalmological products and otic dosage forms that are formulated as suspensions, emulsions or gels) or complex dosage forms (e.g., transdermals, metered dose inhalers, extended release injectables); 2. Complex drug-device combination products (e.g., auto injectors, metered dose inhalers); and 3. Other products where complexity or uncertainty concerning the approval pathway or possible alternative approach would benefit from early scientific engagement (2). Complex generics are generic versions of complex products.

In accordance with the GDUFA, the FDA Office of Generic Drugs (OGD) consults with industry and the public to identify regulatory science initiatives for generic drugs. For example, priority initiatives for fiscal year 2021 include complex active ingredients, formulations, or dosage forms; complex routes of delivery; complex drug-device combination products; and tools and methodologies for PE, BE and therapeutic equivalence (TE) evaluation (3). These priority initiatives reflect categories of complex generic products described in the GDUFA II Commitment Letter (2). This emphasis on complex generics reflects the awareness that, in the years ahead, among other things, expiring patents and exclusivities for several complex products will open opportunities for the availability complex generics, which have the potential to enhance patient access and provide cost savings to the U.S. healthcare system (4, 5).

With funding from FDA, the CRCG established a collaboration between the University of Maryland Baltimore, the University of Michigan, and the FDA. While there have been routine processes (e.g., public meetings, docket input) by which diverse public input could be provided to FDA, the newly formed CRCG sought to understand the concerns and priorities of stakeholders involved in developing complex generics, and a survey was developed so that input from generic industry stakeholders could be provided in a consistent format, which would be amenable to quantitative analysis, and which could provide an objective basis for the CRCG to focus its efforts in areas where specific issues were broadly regarded as priority initiatives.

## SURVEY

In order to appropriately prioritize the CRCG’s initiatives to educate and to conduct research that facilitates the development of complex generic drugs, an online survey entitled “Survey of Scientific Challenges in the Development of Complex Generics” sought input from generic industry stakeholders about which (types of) complex products, which methods of analysis, and which educational topics should be considered a priority for the CRCG’s initial focus areas. Demographic information of respondents was also collected (i.e., current employment or perspective, number of company employees, employer’s interest in complex generics).

The survey was approved by the University of Maryland Baltimore Institutional Review Board. The survey was open to the public on a website maintained by the CRCG (www.complexgenerics.org). The only required answer in the survey was to the consent question. In advancing awareness of the survey, the aim was to receive feedback from a substantial number of pharmaceutical scientists who have an interest in promoting public standards for complex generics. The survey was announced via electronic communications (e.g., on the CRCG website, via emails). Individuals who signed-up to receive CRCG email were notified of the survey. Several organizations created awareness of the survey (e.g., American Association of Pharmaceutical Scientists, Association for Accessible Medicines, International Pharmaceutical Excipients Council of the Americas, Medicines For Europe, Parenteral Drug Association, Pharma & Biopharma Outsourcing Association, Product Quality Research Institute, and Scientists Advancing Affordable Medicines).

Appendix S1 shows the survey. Of note, questions about types of complex products, methods of analysis, and educational topics allowed respondents to select two, two, or four responses, respectively, rather than just one selection. Points of Contact (POCs) were respondents who indicated that they were willing to serve as their institution’s POC (i.e., self-identified as their institution’s Point-of-Contact).

The survey was open from December 3 2020 to February 3 2021. There were 392 responses. One hundred eleven responses were not considered since consent was not provided or no survey answers were provided, resulting in 281 examined responses.

Statistical analysis was performed using SAS (SAS Institute; Cary, NC). A Wald (i.e., 2 × 2) Chi-square test was used to assess differences between POCs *versus* non-POCs in replies about types of complex products, methods of analysis, and educational topics (e.g., Did POCs *versus* non-POCs differ in selecting ophthalmic products as a complex product?).

A Wald Chi-square test was also used to assess differences due to the demographic background of respondents in replies about types of complex products, methods of analysis, and educational topics (e.g., Did generic drug executive or management *versus* all others differ in selecting drug-device combination products as a complex product?). In all cases, no more than 20% of the cells (i.e., entries) had an expected count less than five. P-values from each Wald Chi-square test were examined. To control for multiple comparisons, a Bonferroni correction was applied to p-values, to achieve an overall α = 0.05.

Surveys frequently involve allowance of more than one selection, which is well known to cause statistical limitations, including for a Chi-square test, which assumes all observations are independent from one another and the outcomes are mutually exclusive (6).

## SURVEY DEMOGRAPHICS

The 281 responses consisted of 47 POC respondents and 234 non-POC respondents. Five institutions had more than one employee willing to serve as a POC. Two potential POCs from a regulatory agency were excluded since they were known not to be their institution’s POC. Other potential POCs from institutions with more than one willing POC were excluded if they were not the employee with the highest position (title) among willing POCs from that organization.

Respondents included generic drug industrial scientist or manufacturing personnel (*n* = 51), generic drug executive or management (*n* = 60), or executive or management personnel in a contract research organization (CRO), contract manufacturing organization (CMO), or contract development and manufacturing organization (CDMO) (*n* = 19), innovator drug executive or management (*n* = 15), health care professional (*n* = 11), academic (*n* = 13), and others, for a total of 203 responses. Tables S1a, S1b, and S1c describe the demographics of respondents. In Table S1a, 54.7% of all responses were from generic drug employees, and 12.3% were from CRO, CMO, or CDMO employees. In Table S1b, there were 187 responses, where 47.6% of responses were from employees at companies with at least 1,001 employees. In Table S1c, from 200 responses, 64.0% were a finished dosage form manufacturer, and 26.5% were an active pharmaceutical ingredient (API) manufacturer. Each question about respondent perspective and company size (i.e., Tables S1a and S1b) only allowed one answer. The question about company interests (i.e., Table S1c) allowed multiple selections.

## RANKING OF THE TYPES OF COMPLEX PRODUCTS

Respondents were asked which complex products (excluding biologics) should CRCG focus on now. From a menu of eight options (including “other”), respondents were allowed to select up to two complex product categories (Table I).

**Table I:** Distribution of Replies from Respondents About Complex Products to Focus on Now. Respondents Were Allowed to Select Up to Two Complex Products. Values are Percentages of Respondents Who Selected the specified product. Across all respondents (*n* = 278; 98.9% Response Rate from 281 Survey Respondents), there Were 514 Selections (Average 1.85 Per Respondent, with Range 0–2). Across POCs (*n* = 47; 100% Response Rate From 47 Survey Respondents), There Were 89 Selections (Average 1.89 Per Respondent, With Range 1–2). Across Non-POCs (*n* = 231; 98.7% Response Rate From 234 Survey Respondents), There Were 425 Selections (Average 1.84 Per Respondent, With Range 0–2). POCs and Non-POCs Did Not Differ in any Product Reply (Wald Chi-square *p* > 0.1)

Complex product	All respondents (n = 278)	Points-of-Contact (POCs) (n = 47)	Non-POCs (n = 231)
Complex injectables, formulations, and nanomaterials	54.3%	61.7%	52.8%
Drug-device combination products	29.5%	19.1%	31.6%
Inhalation and nasal products	25.2%	25.5%	25.1%
Long-acting injectables and implants	22.3%	27.7%	21.2%
Complex mixtures and peptides	19.4%	21.3%	19.0%
Topical dermatologic drug products	14.7%	14.9%	14.7%
Ophthalmic products	12.9%	10.6%	13.4%
Other drug or drug product	6.5%	8.5%	6.1%

**Fig. 1:**
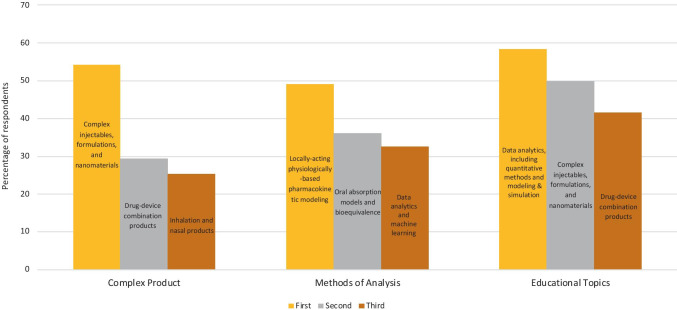
Rank-order of top three replies from all respondents about which complex products, which methods of analysis, and which educational topics to focus on now.

Figure 1 shows the rank-order of the top three replies from all respondents about which complex product categories to focus on now. The top three replies were: complex injectables, formulations, and nanomaterials; drug-device combination products; and inhalation and nasal products. Table II shows the distribution of replies from each type of current employment. Table III shows the distribution of replies from each company size (i.e., number of employees).

**Table II:** Distribution of Replies From Respondents With Differing Employment About Complex Products To Focus on Now. Respondents Were Allowed to Select Up to Two Complex Products, With *n* = 203 Answering Employment Question. Values are Percentages of Respondents Who Selected the Specified Product. The Six Largest Employment Categories are Shown; Across these Respondents (*n* = 169), There Were 330 Selections (Average 1.95 per Respondent). No Employee Type Differed From All Others in Any Product Reply (Wald Chi-square *p* > 0.2). Likewise, in TableS2a, Generic Drug Employees and Non-Generic Drug Employees Did Not Differ in Any Product Reply (Wald Chi-square *p* > 0.05). Nevertheless, Respondents with Differing Employment Showed a Qualitatively Larger Differences in Emphasis for Drug-Device Combination Products. For Example, There was Even a Two-Fold Difference in Emphasis for Drug-Device Combination products between generic drug executive or management (33.3%) *versus* generic drug Industrial Scientist or Manufacturing Personnel (15.7%)

Complex product	Generic drug industrial scientist or manufacturing personnel (*n* = 51)	Generic drug executive or management (*n* = 60)	CRO, CMO, or CDMO executive or management (*n* = 19)	Innovator drug executive or management (*n* = 15)	Health care professional (*n* = 11)	Academic (*n* = 13)
Complex injectables, formulations, and nanomaterials	60.8%	56.7%	52.6%	60.0%	63.6%	61.5%
Drug-device combination products	15.7%	33.3%	21.2%	26.7%	54.5%	38.5%
Inhalation and nasal products	35.3%	26.7%	10.5%	33.3%	36.4%	7.7%
Long-acting injectables and implants	21.6%	21.7%	36.8%	20.0%	0.0%	15.4%
Complex mixtures and peptides	17.6%	15.0%	31.6%	6.7%	9.1%	38.5%
Topical dermatologic drug products	13.7%	15.0%	21.2%	6.7%	9.1%	23.1%
Ophthalmic products	19.6%	8.3%	15.8%	13.3%	9.1%	0.0%
Other drug or drug product	3.9%	6.7%	5.3%	13.3%	9.1%	0.0%

**Table III:** Distribution of Replies from Respondents with Differing Number of Employees About Complex Products to Focus on Now. Respondents (*n* = 187) Were Allowed to Select Up to Two complex products. Values are Percentages of Respondents Who Selected the Specified Product. Across All Respondents (*n* = 187), There Were 354 Selections (Average 1.89 Per Respondent). Companies With More Than 10,000 Employees Differed from All Other Companies in Inhalation and Nasal Products (Wald Chi-square *p* = 0.0002). Meanwhile, in Table S2b, Company Size Categories Differed in Complex Injectables, Formulations, and Nanomaterials (Wald Chi-square *p* = 0.0055)

Complex product	1 (*n* = 10)	2–25 (*n* = 26)	26–100 (*n* = 27)	101–1,000 (*n* = 35)	1,001–10,000 (*n* = 39)	More than 10,000 (*n* = 50)
Complex injectables, formulations, and nanomaterials	50.0%	50.0%%	37.0%	48.6%	64.1%	68.0%
Drug-device combination products	20.0%	26.9%	53.6%	34.3%	28.1%	24.0%
Inhalation and nasal products	30.0%	19.2%	14.8%	20.0%	23.1%	46.0%
Long-acting injectables and implants	40.0%	15.9%	22.2%	20.0%	25.6%	18.0%
Complex mixtures and peptides	30.0%	15.4%	22.2%	14.3%	23.1%	20.0%
Topical dermatologic drug products	10.0%	30.8%	18.5%	20.0%	12.8%	4.0%
Ophthalmic products	0.0%	19.2%	14.8%	11.4%	10.3%	12.0%
Other drug or drug product	20.0%	11.5%	0.0%	11.4%	5.1%	2.0%

Generic drug executives or management provided the same rank-order (Table II) as all respondents (Fig. 1), while respondents at companies with more than 10,000 employees (Table III) ranked inhalation and nasal products ahead of drug-device combination products. Respondents from larger companies tended to prioritize a focus on inhalation and nasal products (Table III). Generic company executives or management (*n* = 60) was the largest responding employment group. Companies with more than 10,000 employees (n = 50) was the largest responding category for company size.

Table I shows the distribution of replies from all respondents including POCs and non-POCs. Among all respondents, the majority (54.3%) selected complex injectables, formulations, and nanomaterials; 29.5% selected drug-device combination products; and 25.2% selected inhalation and nasal products. Complex injectables, formulations, and nanomaterials was consistently identified as the highest priority for focus among types of complex products, by the majority of POCs (61.7%) as well as non-POCs (52.8%). Also, the ranking and relative proportion of responses for Non-POCs (*n* = 231) were closely aligned with those for all respondents (*n* = 278). However, POCs (*n* = 47) ranked long-acting injectables and implants as the second highest priority (27.7%) instead of drug-device combination products, which was ranked as the second highest priority (29.5%) by all respondents.

## RANKING OF METHODS OF ANALYSIS AND TOOLS

Respondents were asked which analytical techniques (implicitly, referring to methods of analysis that would be useful to support a demonstration of bioequivalence) should CRCG focus on now (excluding biologics). From a menu of six options (including “other”), respondents were allowed to select up to two methods of analysis.

Figure 1 shows the rank-order of top three replies from all respondents about methods of analysis. The top three replies were: locally-acting physiologically-based pharmacokinetic modeling; oral absorption models and bioequivalence; and data analytics and machine learning. Generic drug executives and management, as well as respondents at companies with more than 10,000 employees, placed data analytics and machine learning ahead of oral absorption models and bioequivalence.

In Table IV, among all respondents, 49.2% selected locally-acting physiologically-based pharmacokinetic modeling; 36.1% selected oral absorption models and bioequivalence; and 32.5% selected data analytics and machine learning. Table V shows the distribution of replies from each type of current employment. The same methods of analysis were the top three selected by generic drug executive or management. Table VI shows the distribution of replies from respondents at each company size. The larger companies more frequently selected locally-acting physiologically-based pharmacokinetic modeling as a top priority.

**Table IV:** Distribution of Replies From Respondents About Methods of Analysis to Focus on Now. Respondents Were Allowed to Select Up To Two Methods of Analysis. Values are Percentages of Respondents Who Selected the Specified Methods of Analysis. Across All Respondents (*n* = 252; 89.7% Response Rate), There Were 446 Selections (Average 1.77 Per Respondent, With range 0–2). Across POCs (*n* = 47; 100% response rate), there were 86 selections (average 1.83 per Respondent, With Range 1–2). Across Non-POCs (n = 205; 87.6% Response rate), There Were 360 Selections (average 1.76 Per Respondent, With Range 0–2). POCs and Non-POCs Did Not Differ in Any Response Related to Methods of Analysis (Wald Chi-square p > 0.03)

Methods of analysis	All respondents (*n* = 252)	Points-of-Contact (POCs) (*n* = 47)	Non-POCs (*n* = 205)
Locally-acting physiologically-based pharmacokinetic modeling	49.2%	51.1%	48.8%
Oral absorption models and bioequivalence	36.1%	34.0%	36.6%
Data analytics and machine learning	32.5%	36.2%	31.7%
Patient substitution of generic drugs	26.2%	19.1%	27.8%
Quantitative clinical pharmacology	17.9%	19.1%	17.6%
Other analytical techniques and/or drug or drug product	15.1%	23.4%	13.2%

**Table V:** Distribution of Replies from Respondents with Differing Employment About Methods of Analysis To Focus on now. Respondents Were Allowed to Select Up to Two Methods of Analysis, With *n* = 203 Answering Employment Question. Values are Percentages of Respondents Who Selected the Specified Methods of Analysis. The Six Largest Employment Categories are Shown; Across All These Respondents (*n* = 169), There Were 297 Selections (Average 1.76 per Respondent). Generic Drug Industrial Scientist or Manufacturing Personnel Differed From All Others in Locally-Acting Physiologically-Based Pharmacokinetic Modeling (Wald Chi-Square *p* = 0.0004). Generic Drug Executive or Management Differed From All Others in Other (Wald Chi-square *p* = 0.0003). Meanwhile, in Table S3a, Employee Categories Differed in Quantitative Clinical Pharmacology (Wald Chi-square *p* = 0.002)

Methods of analysis	Generic drug industrial scientist or manufacturing personnel (*n* = 51)	Generic drug executive or management (*n* = 60)	CRO, CMO, or CDMO executive or management (*n* = 19)	Innovator drug executive or management (*n* = 15)	Health care professional (*n* = 11)	Academic (*n* = 13)
Locally-acting physiologically-based pharmacokinetic modeling	66.7%	48.3%	31.2%	33.3%	63.6%	23.1%
Oral absorption models and bioequivalence	35.3%	31.7%	36.8%	33.3%	9.1%	46.2%
Data analytics and machine learning	47.1%	36.7%	31.6%	33.3%	27.3%	0.0%
Patient substitution of generic drugs	13.7%	20.0%	26.3%	20.0%	27.3%	53.8%
Quantitative clinical pharmacology	5.9%	11.7%	26.3%	26.7%	54.5%	23.1%
Other analytical techniques and/or drug or drug product	7.8%	28.3%	15.8%	26.7%	9.1%	23.1%

**Table VI:** Distribution of Replies from Respondents with Differing Number of Employees About Methods of Analysis to Focus on Now. Respondents (*n* = 187) Were Allowed to Select Up to Two Methods of Analysis. Values are Percentages of Respondents Who Selected the Specified Methods of Analysis. Across All Respondents (*n* = 187), There Were 333 Selections (Average 1.78 Per Respondent). Companies with More Than 10,000 Employees Differed From All Other Companies in Locally-Acting Physiologically-Based Pharmacokinetic Modeling (Wald Chi-Square *p* = 0.0001). Likewise, in Table S3b, Company Size Categories Differed in Locally-Acting Physiologically-Based Pharmacokinetic Modeling (Wald Chi-Square *p* = 0.0045)

Methods of analysis	1 (*n* = 10)	2–25 (*n* = 26)	26–100 (*n* = 27)	101–1,000 (*n* = 35)	1,001–10,000 (*n* = 39)	More than 10,000 (*n* = 50)
Locally-acting physiologically-based pharmacokinetic modeling	50.0%	50.0%	22.2%	45.7%	43.6%	76.0%
Oral absorption models and bioequivalence	10.0%	26.9%	37.0%	45.7%	33.3%	26.0%
Data analytics and machine learning	50.0%	23.1%	25.9%	25.7%	38.5%	50.0%
Patient substitution of generic drugs	20.0%	26.9%	33.3%	25.7%	28.2%	14.0%
Quantitative clinical pharmacology	20.0%	15.4%	18.5%	20.0%	23.1%	8.0%
Other analytical techniques and/or drug or drug product	20.0%	30.8%	22.2%	14.3%	12.8%	18.0%

## RANKING OF EDUCATIONAL TOPICS

Respondents were asked which educational topics should CRCG focus on now (excluding biologics). From a menu of 13 options (including “other”), respondents were allowed to select up to four educational topics. Potential answers about educational topics were essentially the combined collection of potential answers from the types of complex products and methods of analysis questions.

Figure 1 shows the rank-order of top three replies from all respondents about educational topics. The top three replies were: complex injectables, formulations, and nanomaterials; drug-device combination products; and data analytics, including quantitative methods and modeling & simulation. Generic drug executives and management provided the same rank-order of the same top three educational topics (Table VIII), while respondents at companies with more than 10,000 employees (Table IX) ranked drug-device combination products highest (66.0%) ahead of complex injectables, formulations, and nanomaterials (60.0%).

Table VII shows that, among all respondents, 58.3% selected complex injectables, formulations, and nanomaterials; 50.0% selected drug-device combination products; and 41.7% selected data analytics, including quantitative methods and modeling & simulation as a top priority educational topic. Among POCs, the same proportion of responses (39.1%) were received for long-acting injectables and implants were as were received for selected data analytics, including quantitative methods and modeling & simulation (39.1%). Table VIII shows the distribution of replies from each type of employment. The three methods of analysis listed above (for all respondents) were also the top three methods of analysis selected by generic drug executive or management respondents. Table IX shows the distribution of replies about educational topics from each company size.

**Table VII:** Distribution of Replies From Respondents About Educational Topics to Focus On Now. Respondents Were Allowed to Select Up to Four Educational Topics. Values are Percentages of Respondents Who Selected the Specified Topic. Across All Respondents (*n* = 240; 85.4% Response Rate), There Were 866 Selections (Average 3.61 Per Respondent, With Range 0–4). Across POCs (*n* = 46; 97.9% Response Rate), There Were 166 Selections (Average 3.61 per Respondent, With Range 0–4). Across Non-POCs (*n* = 194; 82.9% Response rate), There Were 700 Selections (average 3.61 Per Respondent, With Range 0–4). POCs and Non-POCs Did Not Differ in Any Topic Reply (Wald Chi-Square p > 0.05)

Educational topic	All respondents (*n* = 240)	Points-of-Contact (POCs) (*n* = 46)	Non-POCs (*n* = 194)
Complex injectables, formulations, and nanomaterials	58.3%	60.9%	57.7%
Drug-device combination products	50.0%	41.3%	52.1%
Data analytics, including quantitative methods and modeling & simulation	41.7%	39.1%	42.3%
Long-acting injectables and implants	31.3%	39.1%	29.4%
Locally-acting physiologically-based pharmacokinetic modeling	29.6%	32.6%	28.9%
Complex mixtures and peptides	26.3%	30.4%	25.3%
Oral absorption models and bioequivalence	25.8%	32.6%	24.2%
Inhalation and nasal products	25.0%	26.1%	24.7%
Patient substitution of generic drugs	20.0%	13.0%	21.6%
Topical dermatologic drug products	19.2%	15.2%	20.1%
Ophthalmic products	19.2%	17.4%	19.6%
Quantitative clinical pharmacology	9.6%	10.9%	9.3%
Other educational topic	4.5%	2.2.%	5.7%

**Table VIII:** Distribution of Replies From Respondents With Differing Employment About Educational Topics to Focus on Now. Respondents Were Allowed to Select Up to Four Educational Topics, with *n* = 203 Answering Employment Question. Values are Percentages of Respondents Who Selected the Specified Educational Topic. The Six Largest Employment Categories are Shown; Across All These Respondents (*n* = 169), There Were 607 Selections (Average 3.59 Per Respondent). No Employee Type Differed From All Others in Any Topic Reply (Wald Chi-square *p* > 0.004). Likewise, in Table S4a, Generic Drug Employees and Non-Generic Drug Employees did Not Differ in Any Topic Reply (Wald Chi-square p > 0.04)

Educational topic	Generic drug industrial scientist or manufacturing personnel (*n* = 51)	Generic drug executive or management (*n* = 60)	CRO, CMO, or CDMO executive or management (*n* = 19)	Innovator drug executive or management (*n* = 15)	Health care professional (*n* = 11)	Academic (*n* = 13)
Complex injectables, formulations, and nanomaterials	68.6%	55.0%	63.2%	53.3%	72.7%	46.2%
Drug-device combination products	52.9%	53.3%	38.8%	40.0%	54.5%	61.5%
Data analytics, including quantitative methods and modeling & simulation	47.1%	46.7%	31.6%	40.0%	45.5%	30.8%
Long-acting injectables and implants	35.3%	21.7%	47.4%	33.3%	18.2%	38.5%
Locally-acting physiologically-based pharmacokinetic modeling	37.3%	36.7%	21.1%	46.7%	36.4%	0.0%
Complex mixtures and peptides	27.5%	23.3%	42.1%	6.7%	18.2%	15.4%
Oral absorption models and bioequivalence	11.8%	23.3%	31.6%	33.3%	9.1%	38.5%
Inhalation and nasal products	23.5%	20.0%	26.3%	40.0%	36.4%	23.1%
Patient substitution of generic drugs	9.8%	18.3%	15.8%	26.7%	18.2%	30.8%
Topical dermatologic drug products	15.7%	18.3%	26.3%	6.7%	27.3%	15.4%
Ophthalmic products	25.5%	23.3%	21.1%	6.7%	0.0%	23.1%
Quantitative clinical pharmacology	5.9%	6.7%	10.5%	6.7%	27.3%	15.4%
Other educational topic	3.9%	5.0%	10.5%	13.3%	0.0%	0.0%

**Table IX:** Distribution of Replies From Respondents with Differing Number of Employees About Educational Topics to Focus on Now. Respondents (*n* = 187) Were Allowed to Select Up to Four Educational Topics. Values are Percentages of Respondents Who Selected the Specified Educational Topic. Across All Respondents (*n* = 187), There Were 676 Selections (Average 3.61 Per Respondent). Companies With More Than 10,000 Employees Differed From All Other Companies in Locally-Acting Physiologically-Based Pharmacokinetic Modeling (Wald Chi-Square *p* = 0.0001), as Well as Data Analytics Including Quantitative Methods and Modeling & Simulation (Wald Chi-square *p* = 0.0004). Meanwhile, in Table S4b, Company Size Categories did not Differ in Any Topic Reply (Wald Chi-square *p* > 0.02)

Educational topic	1 (*n* = 10)	2–25 (*n* = 26)	26–100 (*n* = 27)	101–1,000 (*n* = 35)	1,001–10,000 (*n* = 39)	More than 10,000 (*n* = 50)
Complex injectables, formulations, and nanomaterials	70.0%	69.2%	48.1%	54.3%	64.1%	60.0%
Drug-device combination products	20.0%	42.3%	66.7%	42.9%	43.6%	66.0%
Data analytics, including quantitative methods and modeling & simulation	40.0%	23.1%	40.7%	37.1%	43.6%	58.0%
Long-acting injectables and implants	50.0%	23.1%	22.2%	34.3%	30.8%	28.0%
Locally-acting physiologically-based pharmacokinetic modeling	50.0%	30.8%	3.7%	28.6%	25.6%	54.0%
Complex mixtures and peptides	20.0%	26.9%	25.9%	17.1%	35.9%	20.0%
Oral absorption models and bioequivalence	10.0%	23.1%	18.5%	37.1%	25.6%	18.0%
Inhalation and nasal products	20.0%	26.9%	25.9%	25.7%	23.1%	24.0%
Patient substitution of generic drugs	30.0%	15.4%	33.3%	25.7%	17.9%	8.0%
Topical dermatologic drug products	20.0%	38.5%	18.5%	20.0%	15.4%	8.0%
Ophthalmic products	10.0%	30.8%	22.2%	8.6%	17.9%	22.0%
Quantitative clinical pharmacology	10.0%	7.7%	11.1%	17.1%	10.3%	6.0%
Other educational topic	20.0%	11.5%	3.7%	5.7%	5.1%	2.0%

## HARMONIZED INTERNATIONAL APPROACH

**Fig. 2:**
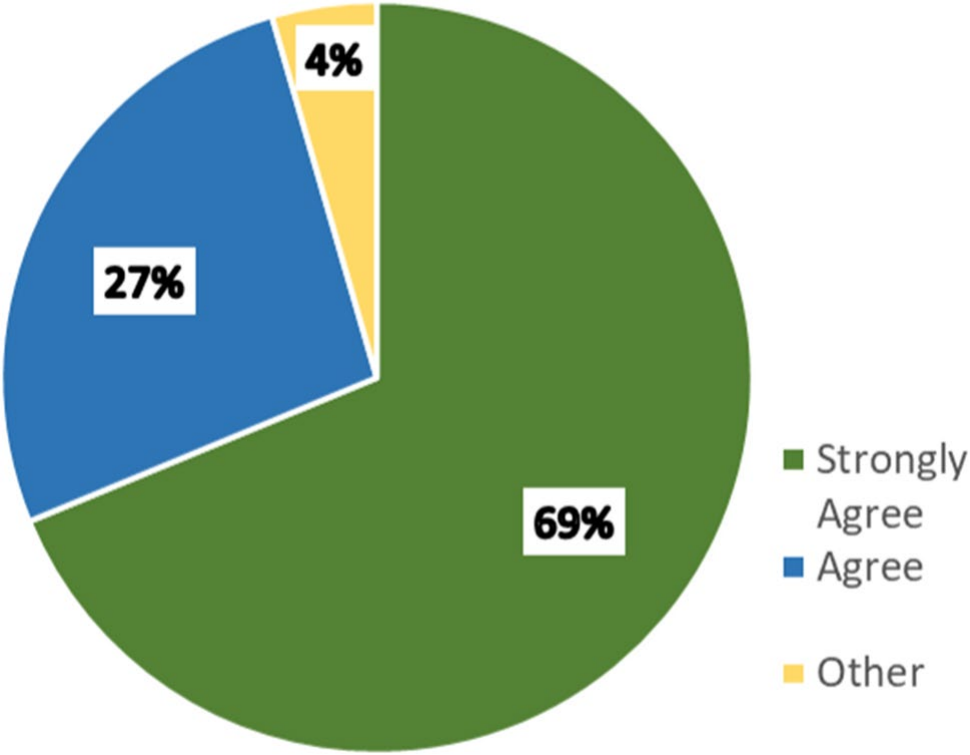
Distribution of replies from all respondents about level of agreement with the statement about the importance of a harmonized international approach for regulatory standards related to the development and approval of complex generic products. Respondents were allowed to select one level of agreement. Table S5 provides greater detail.

Figure 2 illustrates the distribution of replies from all respondents about the level of agreement with a statement about the importance of a harmonized international approach for regulatory standards related to the development and approval of complex generic products (hybrid products in Europe). There was a clear consensus about the importance of global harmonization for complex generic products; 95.5% of respondents agreed or strongly agreed with the statement. A high level agreement was recorded from POCs as well as non-POCs.

## IMPLICATION OF FINDINGS

Survey participation appeared to be very good, with 281 examined responses. Over half of the respondents, including POCs, were generic drug employees. Respondents represented a balanced distribution between companies with at most 1,000 employees and companies with at least 1,001 employees, although most POCs were from larger companies. A majority of respondents were employed by a finished dosage form manufacturer, and one quarter by an API manufacturer. Overall, there was good representation from respondents across all the demographic segments, including different types of generic industry stakeholders, different types of professions, and different sizes of organizations or entities. We believe this survey is the first systematic assessment and analysis of the challenges faced by various stakeholders involved in developing complex generics, and that it provides the first objective classification and ranking of the issues these challenges and of the priorities for addressing them.

There were clear and relatively consistent trends within and across the three main areas of questioning, concerning the types of complex products, methods of analysis, and educational topics on which the CRCG should focus. The results indicate that several areas should be considered priorities for the CRCG’s initial efforts. In Fig. 1, complex injectables, formulations, and nanomaterials, as well as drug-device combination products, scored highest in the responses about the types of complex products and the educational topics of greatest interest. Inhalation and nasal products were also products of major interest, particularly for large organizations with more than 10,000 employees. Data analytics was both, a top method of analysis and a top educational topic. The two top methods of analysis were locally-acting physiologically-based pharmacokinetic modeling, as well as oral absorption models and bioequivalence.

**Fig. 3:**
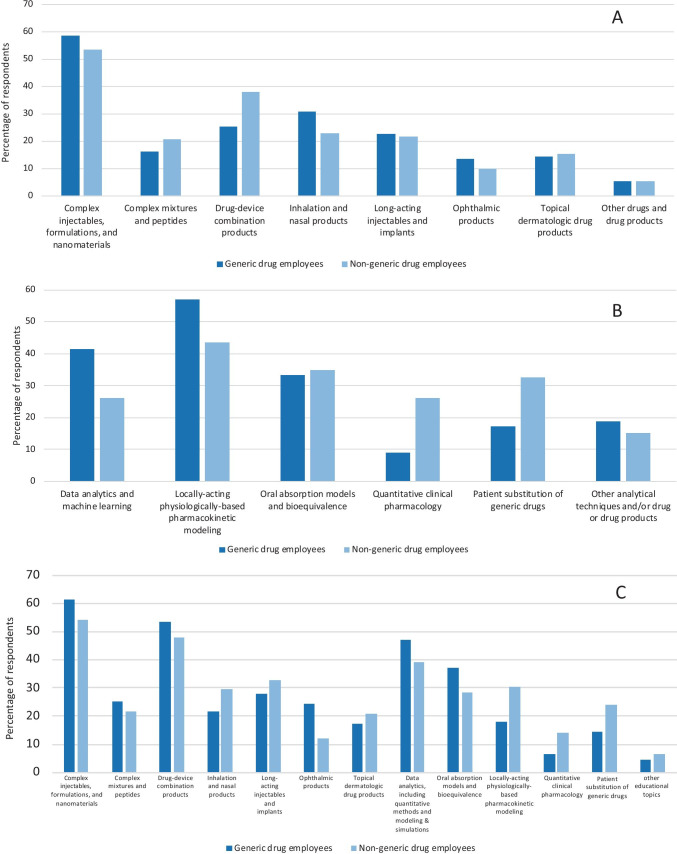
Distribution of replies from generic drug employees and non-generic drug employees about complex products to focus on now (panel A), methods of analysis to focus on now (panel B), and educational topics to focus on now (panel C). Respondents were allowed to select up to two complex products, two methods of analysis, and four education topics, respectively. Values are percentages of respondents who selected the specified type of product, method of analysis, or education topic. Tables S2a, S3a, and S4a list percentage values and greater details.

Regarding types of complex products on which to focus, larger companies tended to emphasize inhalation and nasal products, as well as complex injectables, formulations, and nanomaterials, to a greater extent than smaller companies. Larger companies also emphasized locally-acting physiologically-based pharmacokinetic modeling, as a top method of analysis. Interestingly, although POCs were more frequently from larger companies, POCs and non-POCs provided indistinguishable replies. Likewise, generic drug employees and non-generic drug employees generally provided similar replies, as shown in Fig. 3. There was very strong support for a harmonized international approach for regulatory standards related to the development and approval of complex generic products.

These survey results will help the CRCG prioritize the specific research initiatives and educational programs that will be developed to support generic industry stakeholders in their efforts to develop complex generics. Some survey findings merit further clarification. For example, respondents identified complex injectables, formulations, and nanomaterials as the product category that merited focus now. A clarifying question is: Are there specific methods of analysis and tools for these particular products that merit attention? Respondents also identified data analytics and machine learning as a top method of analysis on which the CRCG should focus now. A clarifying question is: Are there specific applications or product types that may best benefit from data analytics and machine learning?

It is essential that the CRCG continues to engage with generic industry stakeholders to better characterize the specific challenges and to identify actionable outcomes that can facilitate the development and assessment of complex generics. The results presented here indicate that the CRCG should organize scientific workshops focused on the top priority areas for complex generics identified in the survey, like locally-acting physiologically based pharmacokinetic modeling, as well as complex injectables, formulations, and nanomaterials. Such workshops would be ideal forums for education, deliberation, and discussions that can better characterize the challenges for complex generics and potential approaches by which to overcome these challenges in order to enhance patient access to affordable, high quality, complex generic medicines.

## Supplementary Information

Below is the link to the electronic supplementary material.

Supplementary file1 (DOCX 38 kb)

## Abbreviations

API: Active pharmaceutical ingredient
BE: Bioequivalence
CDMO: Contract development and manufacturing organization
CMO: Contract manufacturing organization
CRCG: Center for Research on Complex Generics
CRO: Contract research organization
FDA: Food and Drug Administration
GDUFA: Generic Drug User Fee Amendments
OGD: Office of Generic Drugs
PE: Pharmaceutical equivalence
POC: Point of Contact
TE: Therapeutic equivalence
U.S.: United States
