# Supplementary material for: Research and Education Needs for Complex Generics
Source: Pharm Res. 2021 Dec 24;38(12):1991–2001. doi: 10.1007/s11095-021-03149-y (PMC8732887; doi:10.1007/s11095-021-03149-y)
Supplement: Supplementary file 1 — Supplementary file1 (DOCX 38 kb) [file 11095_2021_3149_MOESM1_ESM.docx]

Supplementary Material for

Research and Education Needs for Complex Generics

Sydney Stern^1^, Jill Coghlan^2^, Vishalakshi Krishnan ^2^, Sam G. Raney^3^, Andrew Babiskin^3^, Wenlei Jiang^3^, Robert Lionberger^3^, Xiaoming Xu^4^, Anna Schwendeman^2^, and James E. Polli^1^

^1^ University of Maryland, Department of Pharmaceutical Sciences, 20 Penn Street, Baltimore, MD 21201, USA

^2^ University of Michigan, Department of Pharmaceutical Sciences, 428 Church Street, Ann Arbor MI 48109, USA

^3^ Food and Drug Administration, Center for Drug Evaluation and Research, Office of Generic Drugs, Office of Research and Standards, 10903 New Hampshire Avenue, White Oak, MD 20993, USA

^4^ Food and Drug Administration, Center for Drug Evaluation and Research, Office of Pharmaceutical Quality, Office of Testing and Research, 10903 New Hampshire Avenue, White Oak, MD 20993, USA

Suggested running head: survey about complex generics

Corresponding author: James E. Polli, Department of Pharmaceutical Sciences, University of Maryland, 20 Penn Street, Baltimore, MD 21201 U.S.A., phone 410-465-3466, jpolli@rx.umaryland.edu

Table of Contents

Table S1a. Distribution of replies from respondents about current employment or perspective.

Table S1b. Distribution of replies from respondents about number of employees in their company.

Table S1c. Distribution of replies from respondents about employer’s interest in complex products or complex generics.

Table S2a. Distribution of replies from generic drug employees and non-generic drug employees about complex products to focus on now.

Table S2b. Distribution of replies from respondents with differing number of employees about complex products to focus on now.

Table S3a. Distribution of replies from generic drug employees and non-generic drug employees about methods of analysis to focus on now.

Table S3b. Distribution of replies from respondents with differing number of employees about methods of analysis to focus on now.

Table S4a. Distribution of replies from generic drug employees and non-generic drug employees about educational topics to focus on now.

Table S4b. Distribution of replies from respondents with differing number of employees about educational topics to focus on now.

Table S5. Distribution of replies from all respondents about level of agreement with the statement about harmonized international approach for complex generics requirements.

Table S1a. Distribution of replies from respondents about current employment or perspective. Respondents were allowed to select one type of employment/perspective. Values are percentages of responses. There were 203 responses (72.2% response rate from 281 survey respondents). 54.7% of all 203 responses were from generic drug employees, and 12.3% were from CRO, CMO, or CDMO employees. Across POCs, there were 45 responses (95.7% response rate from 47 survey respondents). 57.8% of POC responses were from generic drug employees, and 11.1% were from CRO, CMO, or CDMO employees. Across non-POCs, there were 158 responses (56.2% response rate from 234 survey respondents). 53.8% of all non-POC responses were from generic drug employees, and 12.7% were from CRO, CMO, or CDMO employees. POCs and non-POCs did not differ in any employee type reply (Wald Chi-square p>0.08).

| Current employment or perspective | All respondents (n=203) | Points-of-Contact (POCs) (n=45) | Non-POCs (n=158) |
| --- | --- | --- | --- |
| Generic drug industrial scientist or manufacturing personnel | 25.1% (51) | 20.0% | 26.6% |
| Generic drug executive or management | 29.6% (60) | 37.8% | 27.2% |
| CRO, CMO, or CDMO scientist or manufacturing personnel | 3.0% (6) | 2.2% | 3.2% |
| CRO, CMO, or CDMO executive or management | 9.4% (19) | 8.9% | 9.5% |
| Innovator drug industrial scientist or manufacturing personnel | 3.4% (7) | 0.0% | 4.4% |
| Innovator drug executive or management | 7.4% (15) | 11.1% | 6.3% |
| Health care professional | 5.4% (11) | 4.4% | 5.7% |
| Patient or member of the public | 2.0% (4) | 0.0% | 2.5% |
| Health care administrator | 0.0% | 0.0% | 0.0% |
| Regulatory agency employee | 3.9% (8) | 2.2% | 4.4% |
| Non-profit employee | 2.5% (5) | 4.4% | 1.9% |
| Academic | 6.4% (13) | 8.9% | 5.7% |
| Student | 2.0% (4) | 0.0% | 2.5% |

Table S1b. Distribution of replies from respondents about number of employees in their company. Respondents were allowed to select one option. Values are percentages of responses. There were 187 responses (66.6% response rate from 281 survey respondents); 47.6% of responses were from employees at companies with at least 1,001 employees. Across POCs, there were 47 responses (100.0% response rate); 59.6% of responses were from employees at companies with at least 1,001 employees. Across non-POCs, there were 140 responses (59.8% response rate from 234 survey respondents); 43.5% of responses were from employees at companies with at least 1,001 employees. POCs and non-POCs differed in the 101-1,000 and 1,001-10,000 categories (Wald Chi-square p=0.0001 and 0.0002, respectively).

| Number of employees | All respondents (n=187) | Points-of-Contact (POCs) (n=47) | Non-POCs (n=140) |
| --- | --- | --- | --- |
| 1 | 5.3% (10) | 0.0% | 7.1% |
| 2-25 | 13.9% (26) | 2.1% | 17.8% |
| 26-100 | 14.4% (27) | 0.0% | 19.3% |
| 101-1,000 | 18.7% (35) | 38.3% | 12.1% |
| 1,001-10,000 | 20.9% (39) | 31.9% | 17.1% |
| More than 10,000 | 26.7% (50) | 27.7% | 26.4% |

Table S1c. Distribution of replies from respondents about employer’s interest in complex products or complex generics. Respondents were allowed to select any number of interests. Values are percentages of respondents who selected the specified interest. Across all respondents (n=200; 71.2% response rate), there were 360 selections (average 1.80 per respondent, with range 0-8). Across POCs (n=47; 100% response rate), there were 106 selections (average 2.26 per respondent, with range 1-8). Across non-POCs (n=153; 65.4% response rate), there were 254 selections (average 1.66 per respondent, with range 0-8). From all respondents, 64.0% were a finished dosage form manufacturer, and 26.5% were an active pharmaceutical ingredient (API) manufacturer. POCs and non-POCs differed in the API manufacturer, finished dosage form manufacturer, packaging, and CRO-other R&D categories (Wald Chi-square p=0.0003, 0.0001, 0.002, and 0.001, respectively).

| Employer’s interest in complex products or complex generics | All respondents (n=200) | Points-of-Contact (POCs) (n=47) | Non-POCs (n=153) |
| --- | --- | --- | --- |
| API manufacturer | 26.5% | 38.3% | 22.9% |
| Finished dosage form manufacturer | 64.0% | 72.3% | 61.4% |
| Packaging | 16.5% | 25.5% | 13.7% |
| Excipient manufacturer/provider | 5.5% | 8.5% | 4.6% |
| CRO-analytical | 13.0% | 14.9% | 12.4% |
| CRO-clinical | 10.0% | 14.9% | 8.5% |
| CRO-other R&D | 16.0% | 25.5% | 13.1% |
| Academic | 8.5% | 10.6% | 7.8% |
| Government | 5.0% | 4.3% | 5.2% |
| Health care practitioner | 2.0% | 2.1% | 2.0% |
| Other | 13.0% | 8.5% | 14.4% |

Table S2a. Distribution of replies from generic drug employees and non-generic drug employees about complex products to focus on now. Respondents were allowed to select up to two complex products, with n=203 answering employment question. Values are percentages of respondents who selected the specified product. Across all respondents (n=203), there were 379 selections (average 1.87 per respondent). Across generic drug employees (n=111), there were 207 selections (average 1.86 per respondent). Across non-generic drug employees (n=92), there were 172 selections (average 1.87 per respondent). Generic drug employees consisted of generic drug industrial scientist or manufacturing personnel, or generic drug executive or management. Non-generic drug employee consisted of all others (e.g. CRO employees, innovator drug employees, health care professionals). Generic drug employees and non-generic drug employees did not differ in any product reply (Wald Chi-square p>0.05).

| Complex product | Generic drug employee (n=111) | Non-generic drug employee (n=92) |
| --- | --- | --- |
| Complex injectables, formulations, and nanomaterials | 58.6% | 53.3% |
| Complex mixtures and peptides | 16.2% | 20.7% |
| Drug-device combination products | 25.2% | 38.0% |
| Inhalation and nasal products | 30.6% | 22.8% |
| Long-acting injectables and implants | 22.5% | 21.7% |
| Ophthalmic products | 13.5% | 9.8% |
| Topical dermatologic drug products | 14.4% | 15.2% |
| Other drug or drug product | 5.4% | 5.4% |

Table S2b. Distribution of replies from respondents with differing number of employees about complex products to focus on now. Respondents were allowed to select up to two complex products, with n=187 answering company size question. Values are percentages of respondents who selected the specified product. Across all respondents (n=187), there were 354 selections (average 1.89 per respondent). Across smaller company size (i.e. 1,000-and-less) respondents (n=98), there were 182 selections (average 1.86 per respondent). Across larger company size (i.e. more than 1,000) respondents (n=89), there were 172 selections (average 1.93 per respondent). Company size categories differed in complex injectables, formulations, and nanomaterials (Wald Chi-square p=0.0055).

| Complex product | 1,000-and-less (n=98) | More than 1,000 (n=89) |
| --- | --- | --- |
| Complex injectables, formulations, and nanomaterials | 45.9% | 66.3% |
| Complex mixtures and peptides | 18.4% | 21.3% |
| Drug-device combination products | 36.7% | 25.8% |
| Inhalation and nasal products | 19.3% | 40.0% |
| Long-acting injectables and implants | 21.4% | 21.3% |
| Ophthalmic products | 13.3% | 11.2% |
| Topical dermatologic drug products | 21.4% | 7.9% |
| Other drug or drug product | 9.2% | 3.4% |

Table S3a. Distribution of replies from generic drug employees and non-generic drug employees about methods of analysis to focus on now. Respondents were allowed to select up to two a methods of analysis, with n=203 answering employment question. Values are percentages of respondents who selected the specified methods of analysis. Across all respondents (n=203), there were 360 selections (average 1.77 per respondent). Across generic drug employees (n=111), there were 196 selections (average 1.77 per respondent). Across non-generic drug employees (n=92), there were 164 selections (average 1.78 per respondent). Employee categories differed in quantitative clinical pharmacology (Wald Chi-square p=0.002).

| Methods of analysis | Generic drug employee (n=111) | Non-generic drug employee (n=92) |
| --- | --- | --- |
| Data analytics and machine learning | 41.4% | 26.1% |
| Locally-acting physiologically-based pharmacokinetic modeling | 56.8% | 43.5% |
| Oral absorption models and bioequivalence | 33.3% | 34.8% |
| Quantitative clinical pharmacology | 9.0% | 26.1% |
| Patient substitution of generic drugs | 17.1% | 32.6% |
| Other analytical techniques and/or drug or drug product | 18.9% | 15.2% |

Table S3b. Distribution of replies from respondents with differing number of employees about methods of analysis to focus on now. Respondents were allowed to select up to two methods of analysis, with n=187 answering company size question. Values are percentages of respondents who selected the specified methods of analysis. Across all respondents (n=187), there were 333 selections (average 1.78 per respondent). Across smaller company size (i.e. 1,000-and-less) respondents (n=98), there were 160 selections (average 1.63 per respondent). Across larger company size (i.e. more than 1,000) respondents (n=89), there were 173 selections (average 1.94 per respondent). Company size categories differed in locally-acting physiologically-based pharmacokinetic modeling (Wald Chi-square p=0.0045).

| Methods of analysis | 1,000-and-less (n=98) | More than 1,000 (n=89) |
| --- | --- | --- |
| Data analytics and machine learning | 27.6% | 43.8% |
| Locally-acting physiologically-based pharmacokinetic modeling | 40.8% | 61.8% |
| Oral absorption models and bioequivalence | 26.5% | 38.2% |
| Quantitative clinical pharmacology | 18.4% | 14.6% |
| Patient substitution of generic drugs | 27.6 | 20.2% |
| Other analytical techniques and/or drug or drug product | 21.4% | 15.7% |

Table S4a. Distribution of replies from generic drug employees and non-generic drug employees about educational topics to focus on now. Respondents were allowed to select up to four educational topics, with n=203 answering employment question. Values are percentages of respondents who selected the specified methods of analysis. Across all respondents (n=203), there were 571 selections (average 2.81 per respondent). Across generic drug employees (n=111), there were 329 selections (average 2.96 per respondent). Across non-generic drug employees (n=92), there were 242 selections (average 2.63 per respondent). Generic drug employees and non-generic drug employees did not differ in any topic reply (Wald Chi-square p>0.04).

| Educational topic | Generic drug employee (n=111) | Non-generic drug employee (n=92) |
| --- | --- | --- |
| Complex injectables, formulations, and nanomaterials | 61.3% | 54.3% |
| Complex mixtures and peptides | 25.2% | 21.7% |
| Drug-device combination products | 53.2% | 47.8% |
| Inhalation and nasal products | 21.6% | 29.3% |
| Long-acting injectables and implants | 27.9% | 32.6% |
| Ophthalmic products | 24.3% | 12.0% |
| Topical dermatologic drug products | 17.1% | 20.7% |
| Data analytics, including quantitative methods and modeling & simulation | 46.8% | 39.1% |
| Locally-acting physiologically-based pharmacokinetic modeling | 36.9% | 28.3% |
| Oral absorption models and bioequivalence | 18.0% | 30.4% |
| Quantitative clinical pharmacology | 6.3% | 14.1% |
| Patient substitution of generic drugs | 14.4% | 23.9% |
| Other educational topic | 4.5% | 6.5% |

Table S4b. Distribution of replies from respondents with differing number of employees about educational topics to focus on now. Respondents were allowed to select up to four educational topics. Values are percentages of respondents who selected the specified educational topic. Across all respondents (n=187), there were 676 selections (average 3.61 per respondent). Across smaller company size (i.e. 1,000-and-less) respondents (n=98), there were 348 selections (average 3.55 per respondent). Across larger company size (i.e. more than 1,000) respondents (n=89), there were 328 selections (average 3.69 per respondent). Company size categories did not differ in any topic reply (Wald Chi-square p>0.02).

| Educational topic | 1,000-and-less (n=98) | More than 1,000 (n=89) |
| --- | --- | --- |
| Complex injectables, formulations, and nanomaterials | 58.1% | 61.8% |
| Complex mixtures and peptides | 21.4% | 27.0% |
| Drug-device combination products | 46.9% | 56.2% |
| Inhalation and nasal products | 25.5% | 23.6% |
| Long-acting injectables and implants | 29.6% | 29.2% |
| Ophthalmic products | 18.4% | 20.2% |
| Topical dermatologic drug products | 24.5% | 11.2% |
| Data analytics, including quantitative methods and modeling & simulation | 35.0% | 51.7% |
| Locally-acting physiologically-based pharmacokinetic modeling | 24.5% | 41.3% |
| Oral absorption models and bioequivalence | 25.5% | 21.3% |
| Quantitative clinical pharmacology | 12.2% | 7.9% |
| Patient substitution of generic drugs | 25.5% | 12.4% |
| Other educational topic | 8.2% | 3.4% |

Table S5. Distribution of replies from all respondents about level of agreement with the statement about harmonized international approach for complex generics requirements. Respondents were allowed to select one level of agreement. Across all respondents (n=281), there were 243 responses (86.5% response rate), with 95.5% of responses being strongly agree or agree. Across POCs (n=47), there were 46 responses (97.9% response rate), with 97.8% of responses being strongly agree or agree. Across non-POCs (n=234), there were 197 responses (84.2% response rate), with 94.9% of responses being strongly agree or agree.

| Level of agreement | Percentages of responses |
| --- | --- |
| Strongly agree | 68.7% |
| Agree | 26.7% |
| Disagree | 3.3% |
| Strongly disagree | 0.0% |
| Unable to judge | 1.2% |
